# Water deficit and salinity stresses modulate growth, physiology, and phytochemical composition of *Lavandula coronopifolia* Poir. grown in pots under controlled conditions

**DOI:** 10.3389/fpls.2025.1719460

**Published:** 2026-01-07

**Authors:** Hafsa Debbagh-Nour, Ayoub El Mouttaqi, Karima Lazaar, Ihssane Mnaouer, Sanaa Malki, Marc Ducousso, Hassan Boukcim, Abdelaziz Hirich

**Affiliations:** 1 African Sustainable Agriculture Research Institute (ASARI), College of Agriculture & Environmental Sciences (CAES), Mohammed VI Polytechnic University (UM6P), Laayoune, Morocco; 2 CIRAD, UMR AGAP-Institut, Montpellier, France; 3 VALORHIZ SAS, Montpellier, France

**Keywords:** *Lavandula coronopifolia* Poir., water deficit, salinity, oxidative stress, phenolic compounds, antioxidant activity

## Abstract

*Lavandula coronopifolia* Poir. is a medicinal evergreen shrub, wildly distributed in rocky and arid environments. It belongs to the *Lamiaceae* family, known by the large array of bioactive compounds it contains. Drought and salinity present major threats in arid zones and severely penalize the potential yield of naturally growing desertic plants; however, it may affect the synthesis and accumulation of their metabolites. Few studies have investigated the response of *Lavandula* species to abiotic stresses and to the best of our knowledge, none have been conducted on *L. coronopifolia*. Our study aims to investigate various responses of this species to water deficit and salt stress under controlled conditions. Two distinct experiments were conducted in a growth chamber, each lasting one month. The first one focused on water stress, with plants subjected to four water treatments: control (100% field capacity (FC)), moderate water deficit (50% FC), severe water stress (25% FC), and very severe water stress (alternating irrigation to 50% FC for one week followed by cessation of watering for the next week). These treatments were arranged in a randomized complete block design (RCBD) with 3 blocks, each containing 3 replicates per treatment, resulting in 9 replications per treatment. The second experiment investigated the effect of salt stress, where plants were exposed to four NaCl concentrations: 0, 5, 10, and 20 dS/m. This experiment was also conducted using an RCBD, with 4 blocks and 4 replicates per treatment within each block, giving a total of 16 repetitions per treatment. Growth parameters, oxidative stress indicators as well as secondary metabolite content were determined. Results have shown that under both water and salt stress conditions, plant fresh and dry weights decreased significantly. Malondialdehyde levels increased under intense stress in both experiments, indicating enhanced lipid peroxidation. Protein content increased under water stress but showed no change under salt stress. Phenolic and flavonoid contents increased with water stress but decreased with salt stress. Antioxidant activity remained stable under water stress and showed a significant increase with salt stress. These findings enhance our understanding of how plants modulate various traits in response to distinct water and salt stress conditions.

## Introduction

In arid and semi-arid regions, plant species are exposed to a number of biotic and abiotic stresses that severely penalize their performance and their growth potential (Nareshkumar et al., 2020). Among these, drought and salinity present major ecological limitation and environmental challenge.

Salinity affects approximately 1.38 billion hectares of land worldwide, with an additional 1 billion hectares at risk due to climate change and poor land management (FAO, 2024). Water quality is also afflicted, total soluble salts are present in high concentrations due to the overuse of groundwater but also to the intrusion of seawater in some cases (Alfarrah and Walraevens, 2018). Southern regions of Morocco are no exception, more than 14% of soils are very strongly saline with an electrical conductivity higher than 16 dS/m. Drought affects lands across the world, including southern regions of Morocco. It arises mainly from limited water availability combined with high temperature, sun exposure and wind (Xu et al., 2010).

Both stresses cause damage that induces the activation of the plant defense mechanisms on different levels: physiological, phytochemical, and molecular. The alteration of the metabolic system of the cell induces osmotic and oxidative stress leading to an excessive formation of reactive oxygen species (ROS) (Zehra et al., 2019). These include radicals such as hydrogen peroxide, superoxide and hydroxyl, which play a role in signaling and regulating biological processes throughout the plant life cycle (Miller et al., 2008). However, water and salt stress generally have a significant positive impact on the accumulation of osmolytes responsible for osmotic adjustment of plants, a principal role in plant defense response. In parallel, the synthesis of enzymatic and non-enzymatic antioxidant systems is triggered, resulting in higher concentrations of these metabolites. Among these, phenols and flavonoids stand out not only for their crucial role in plant stress tolerance but also for their well-known beneficial properties implemented in the pharmacological field, making them highly desirable, especially in aromatic and medicinal plants (Kapoor et al., 2020; Shil and Dewanjee, 2022).

*Lavandula* is a genus of over 40 known species and around 400 varieties, belonging to the *Lamiaceae* family (Upson and Andrews, 2004). It is native to the Mediterranean basin, East Africa, the Middle East, Southwest Asia and Southeast India, but is currently cultivated in many parts of the world (Lis-Balchin, 2002; Mokhtarzadeh et al., 2013; Dobros et al., 2022) where favorable conditions arise: warm weather, cold winters and sunny summers (Lis-Balchin, 2002). *Lavandula* is widely used in folk medicine to treat many diseases but also in cosmetics and pharmaceutics thanks to the multiple qualities of its essential oil (Gámez et al., 1987; Cavanagh and Wilkinson, 2002; Bouyahya et al., 2023).

Wild lavender (*Lavandula coronopifolia* Poir.), locally known as “كحيلة الخيل “ in southern regions of Morocco, is a medicinal evergreen shrub reaching up to 80 cm (Lis-Balchin, 2002). In Morocco, it naturally grows notably in rocky environments around wadis of Es-Semara and Aousserd provinces known for their arid climate. *Lavandula coronopifolia* is essentially used by indigenous people in the south of Morocco as a medicinal plant against asthma and renal colic. It has been reported to possess several properties including antioxidant (Messaoud et al., 2012), antimicrobial (Hassan et al., 2014), larvicidal and antibacterial (Emam et al., 2021) activities. This species was chosen not only for its medicinal and ecological potential but also to promote its valorization and conservation, as it is not yet domesticated and is threatened in its natural habitats. We hypothesize that water and salt stress will induce distinct but overlapping physiological changes and differentially affect the accumulation of phenolic and flavonoid compounds in *L. coronopifolia*.

Very few studies (Kulak, 2020; Szekely-Varga et al., 2020b; Kumlay et al., 2022; Saunier et al., 2022; Mansinhos et al., 2024) have investigated the response of *Lavandula* species to abiotic stresses and so far, and to the best of our knowledge, none have been conducted on *L. coronopifolia*. Therefore, this study aims to evaluate the effects of water deficit and salinity on the growth, physiological performance, oxidative stress, and phytochemical compositions of *L. coronopifolia*. We hypothesize that these stresses will elicit distinct adaptive responses, providing insights into the resilience and metabolic plasticity of this undomesticated species.

## Material and methods

### Plant material, experimental design, and treatments

Seeds of *L. coronopifolia* were collected at maturity from their natural habitat in Oued Jehch, Haouza, Es-Semara province (27.05200, -11.32083) in March 2021 and were stored at 4°C in a cold room at the African Sustainable Agriculture Research Institute in Laayoune (ASARI). They were multiplied in a growth chamber (Microclima MC1000, Snijders Lab, Netherlands) at 25/20 °C day/night temperatures, 60% of relative humidity, a 14h photoperiod, and a light intensity of 250 µmol/m²/s provided by standard white lamps. CO_2_ levels were maintained at atmospheric values (not monitored by the equipment). Resulting seeds were sown in seed trays. After two months of growth, seedlings were transferred to plastic perforated pots (320 mL) filled with a mixture of sand and peat to a 2:1 ratio and were placed in the growth chamber under the same conditions mentioned above.

Water stress was evaluated after one month of exposure, using a randomized complete block design (RCBD) with 3 blocks, each containing 3 replicates per treatment, resulting in 9 experimental units per treatment. The irrigation regimes supplied 100%, 50%, 25%, and an alternating 0/50% of field capacity (FC), corresponding to control, moderate water stress, severe water stress, and very severe water stress, respectively. The 0/50% FC treatment involved weekly cycles of withholding followed by rewatering at 50% FC. Water was supplied manually three times per week, and the pots were weighed before each irrigation. The amount of water added was adjusted to bring each pot to its target field capacity, ensuring precise control of substrate moisture. For the alternating 0/50% FC treatment, water was withheld for one week, followed by rewatering to 50% FC, with the cycle repeated weekly. These procedures ensured that stress levels were quantitatively maintained throughout the experiment.

To assess the effect of salinity, four NaCl concentrations (0, 5, 10 and 20 dS/m, corresponding approximately to 0, 85, 170, and 340 mM NaCl) were applied through irrigation in a separate RCBD with 4 blocks, each containing 4 replicates per treatment, giving 16 experimental units per treatment. The NaCl concentration was increased gradually to avoid osmotic shock. Plants were irrigated with corresponding solutions until saturation, three times a week. The applied NaCl solutions were intended to induce combined osmotic and ionic stress.

A half-strength Hoagland nutrient solution was prepared and mixed with the irrigation water. It was applied at the same rate for all treatments throughout both the water stress and salinity experiments to ensure uniform nutrient availability.

### Shoot fresh and dry weight

Aerial parts of all samples were harvested at the end of the experiment - one month after stress application- and were weighed. The dry biomass was determined after plants were dried in an oven at 45 °C up to constant weight.

### Oxidative stress analyses

#### Extract preparation

Fresh leaf tissue (250 mg) was homogenized in 4 mL of 50 mM phosphate-buffered saline (PBS), prepared using distilled water along with sodium phosphate dibasic (Na_2_HPO_4_) and sodium phosphate monobasic (NaH_2_PO_4_). The resulting homogenate was centrifuged at 15,000 rpm for 10 minutes at 4 °C. The supernatant obtained was collected and stored at −20 °C for use in subsequent assays.

#### Soluble proteins assessment

Soluble protein content in leaf samples was determined using the Bradford assay (He, 2011), a colorimetric method that relies on the interaction between Coomassie Brilliant Blue G-250 dye and proteins. Upon binding, the dye undergoes a color change from red to blue, with the intensity correlating to protein concentration. Absorbance was measured at 595 nm using a UV-Visible spectrophotometer. Bovine serum albumin (BSA) served as the standard for the calibration curve.

#### Malondialdehyde quantification

Lipid peroxidation in leaf tissues was assessed using the thiobarbituric acid (TBA) assay, which quantifies malondialdehyde (MDA) levels as an indicator of oxidative damage (Buege and Aust, 1978). Absorbance was measured at 532 nm, with background correction performed by subtracting non-specific absorbance at 600 nm. The concentration of the MDA–TBA complex was determined using a molar extinction coefficient of 155 mM^-^¹ cm^-^¹ at 532 nm.

### Phytochemical analyses

#### Extract preparation

Aerial parts of *L. coronopifolia* were washed, dried then ground. Five grams of each sample were extracted by maceration with 50 mL of an aqueous solution of methanol 70% for 24 hours with permanent agitation following a modified protocol of De la Luz Cádiz‐Gurrea et al (De la Luz Cádiz-Gurrea et al., 2019). Extracts were centrifuged and filtered twice through Dorsan filter paper (10 µm pore size) to ensure clarity. The solvent was evaporated using a rotary evaporator (Büchi R-100, Switzerland). Crude extracts were reconstituted in a solution of acidified water and methanol (50:50, v/v) at a concentration of 1 mg/ml for spectrophotometric quantification and 10 mg/mL for HPLC analysis. Extracts were re-filtered with nylon syringe filters (0.22 μm pore size) right before HPLC analysis.

#### Estimation of total phenolic content

The phenolic content was determined following the Folin-Ciocalteu spectrophotometric method as defined by (Lachman et al., 1998), with minor modifications, which consists of the formation of a blue coloration due to the reduction in alkaline medium of the phospho-tungstic and phospho-molybdic mixture of Folin’s reagent by the oxidizable groups of phenolic compounds. The absorbance of the resulting coloration was measured at 765 nm with a UV-Vis spectrophotometer. The calibration curve was prepared using gallic acid as a standard. The final phenolic values are expressed in gallic acid equivalents per gram of dry weight (mg GAE/g DW).

#### Estimation of total flavonoid content

As mentioned by Dinis et al (Dinis et al., 1994), flavonoid content was measured spectrophotometrically using the aluminum chloride method. The quercetin (Sigma-Aldrich) was used as a standard and the measurements of the absorbance were done at 415 nm using a UV-Vis spectrophotometer. Results of calculations were expressed in milligrams of quercetin equivalents per gram of the dry weight (mg QE/g DW).

#### Total antioxidant activity assessment

The determination of the antioxidant activity was done using the DPPH (1,1- diphenyl-2-picrylhydrazyl) free radical scavenging method (Romulo, 2020). The ascorbic acid was used as a positive control, and the absorbance was estimated at 517 nm using a UV-Vis spectrophotometer (VWR, UV-6300PC). The percentage of inhibition of DPPH free radicals was calculated using the following equation:


Inhibition %=[(Abs C–Abs S)/Abs C]×100

With Abs C representing the absorbance of the control and Abs S the absorbance of the extract.

#### Chromatographic conditions

An HPLC instrument (Agilent 1290 Infinity II, Agilent technologies, Germany) equipped with a diode array detector (DAD) was used. Methanol, formic acid, and acetonitrile were of HPLC grade. Ultrapure water was purified by Milli-Q IQ 7000 system (Merck, Germany). Commercial standards of polyphenols and flavonoids are listed below:

Phenolic acids: p-Salicylic acid, Vanillic acid, Caffeic acid, p-Coumaric acid, Sinapinic acid and Ellagic acid.

Flavonoids: Epicatechin and naringenin

The HPLC-DAD was used to identify some phenolic and flavonoid constituents of *L. coronopifolia* aerial parts. Compound identification was confirmed during method development by injecting analytical standards individually and in mixtures to verify their retention times.

For phenolic acids, the separation was performed using an InfinityLab Poroshell 120 EC C18 (2.1 × 100 mm, 2.7 µm) column with an injection volume of 1 µL, a flow rate of 0.3 mL/min and an analysis time of 23 minutes. The mobile phase consisted of two eluants 0.1% formic acid (A) and methanol HPLC grade (B) with gradient elution. Detection was carried out at wavelengths of 254, 270, 308, and 324 nm.

For flavonoids, the analysis was conducted under the same injection volume and flow rate conditions (1 µL, 0.3 mL/min), but using a Zorbax Extend C18 column (2.1 × 50 mm, 1.8 µm) with an analysis time of 17 minutes. The mobile phase consisted of 0.1% formic acid (A) and acetonitrile (B), also with gradient elution, and detection was performed at 200 nm.

The key validation parameters of the method including retention times, linearity (R²), LOD, LOQ, and recovery are presented in **Table 1**.

**Table 1:** HPLC-DAD validation parameters for phenolic acids and flavonoids.

Phenolic acids	Retention time	R²	LoD (ppm)	LoQ (ppm)	Recovery
p-Salicylic acid	8.67	0.9989	1.13	3.42	97.1
Vanillic Acid	11.62	0.9999	0.33	1.00	98.8
Caffeic Acid	12.05	0.9999	0.28	0.85	99.1
p-Coumaric Acid	15.14	0.9999	0.37	1.13	98.9
Sinapinic Acid	16.72	0.9999	0.30	0.91	98.9
Ellagic Acid	18.20	0.9997	0.59	1.79	97.6
Flavonoids	Retention time	R²	LoD (ppm)	LoQ (ppm)	Recovery
Epicatechin	4.651	0.999873	0.36	1.09	101.8
Naringenin	7.743	0.999988	0.12	0.37	100.6

### Data management and statistical analysis

All statistical analyses were performed in the R environment (version 4.4.0 (2024-04–24 ucrt)) within RStudio (version 2024.4.2.764). Data were analyzed using a linear model appropriate for a randomized complete block design (RCBD), with treatment as a fixed factor and block as a random factor. When significant differences were detected (p < 0.05), *post hoc* pairwise comparisons were performed using Tukey’s Honest Significant Difference (HSD) test *via* the “multcomp” package. Group differences were visualized using compact letter displays generated with the “multcompView” package. All plots were created using the “ggplot2” package. Principal Component Analysis (PCA) was performed using the “FactoMineR” and “factoextra” packages to explore trait correlations and treatment effects. Analyses were conducted on raw individual replicate values and performed separately for drought and salinity treatments. The first two components were retained for biplot visualization, and PCA loadings for all variables were provided.

## Results

### Shoot fresh and dry weight

**Figure 1:**
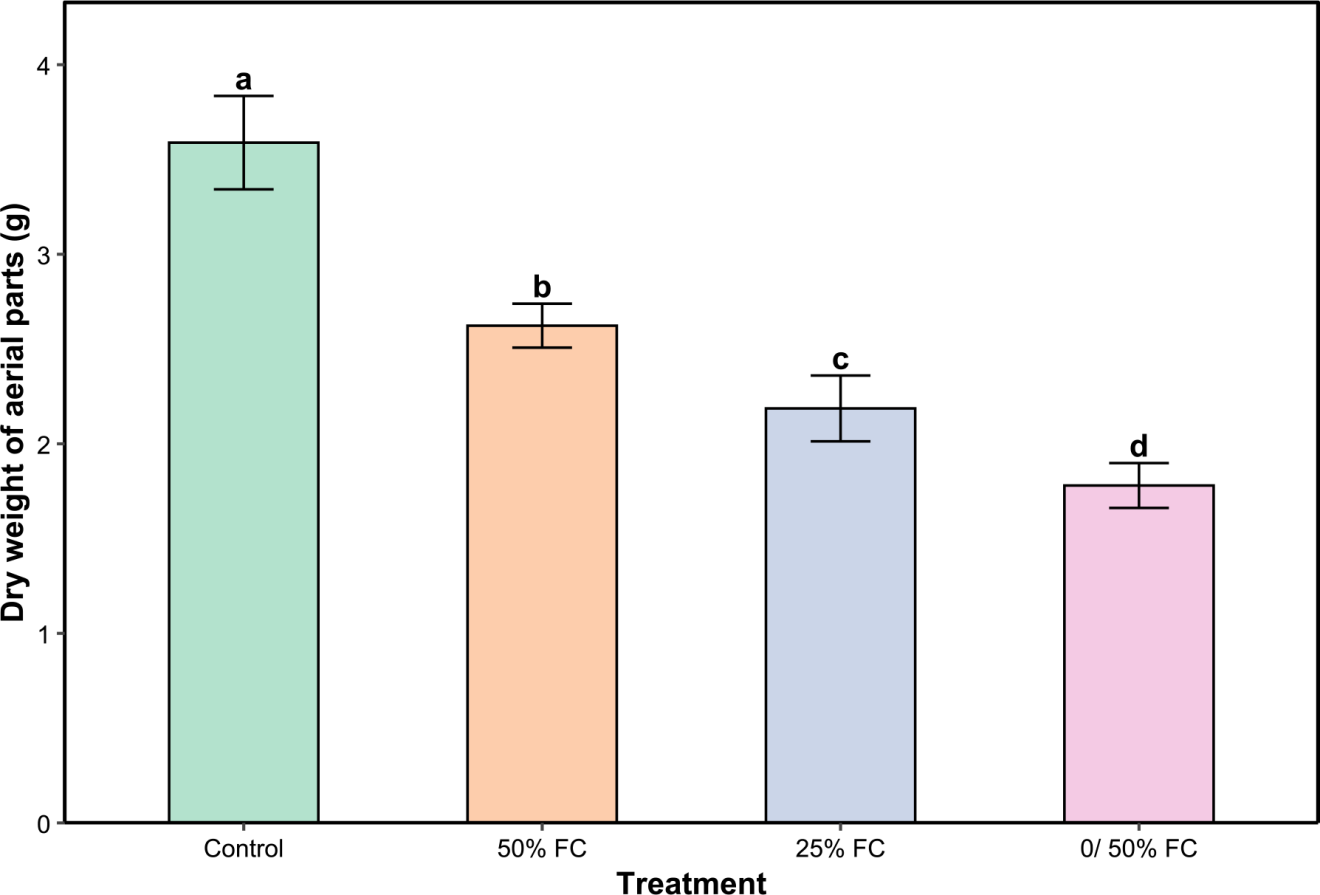
Dry weight of aerial parts of *Lavandula coronopifolia* under water stress treatments. Error bars show the mean ± standard error (n = 9 for water stress and n = 16 for salinity treatments). Significant differences at p < 0.05 level of significance are indicated by different letters above each bar. *0/50% FC: alternating 0% and 50% field capacity weekly..

For water stress, dry to fresh biomass ratio of aerial parts was constant across all treatments (100, 50, 25 and 0/50% FC). Compared to the control, dry weight decreased with increasing stress by 22.45%, 37.42%, and 50% under moderate, severe, and very severe water stress, respectively (**Figure 1**). The most pronounced reductions were observed under very severe water stress compared to the control.

**Figure 2:**
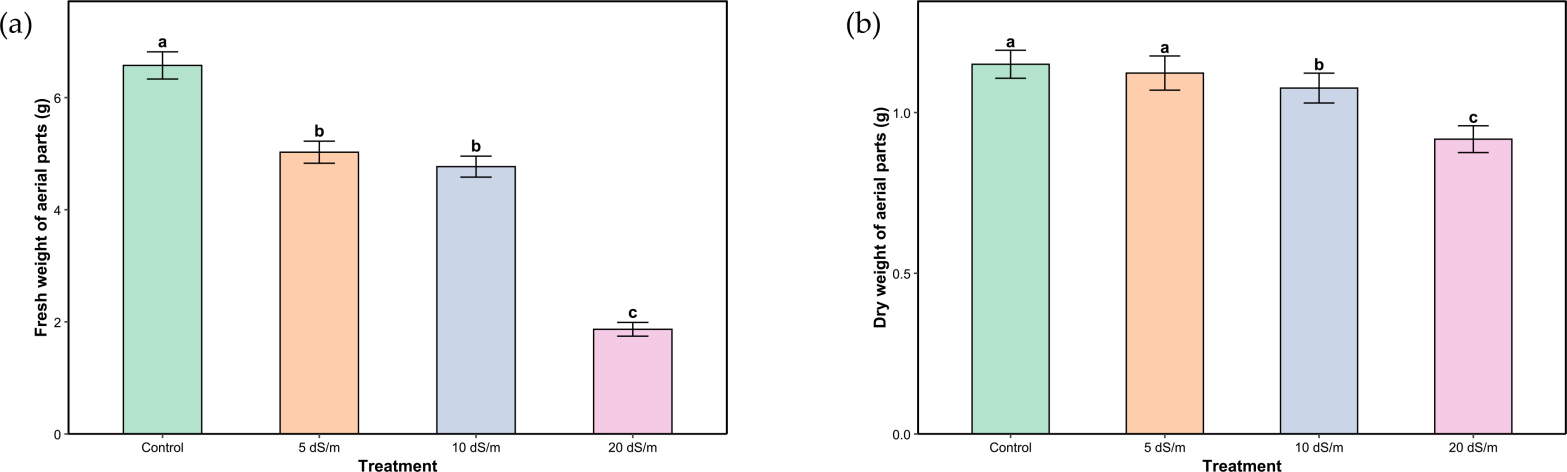
Fresh **(a)** and dry **(b)** weight of aerial parts of *Lavandula coronopifolia* under salt stress treatments. Error bars show the mean ± standard error (n = 9 for water stress and n = 16 for salinity treatments). Significant differences at p < 0.05 level of significance are indicated by different letters above each bar. *0/50% FC: alternating 0% and 50% field capacity weekly.

In the case of salt stress, the proportion of dry matter to fresh weight was not stable suggesting a major shift in water balance and tissue composition, possibly due to dehydration. Fresh weight decreased by 23.44%, 27.40%, and 71.54% under moderate, severe, and very severe stress, respectively, while dry weight dropped by 2.6%, 6.09%, and 23% for the corresponding treatments (**Figures 2A, B**).

### Oxidative stress indicators

**Figure 3:**
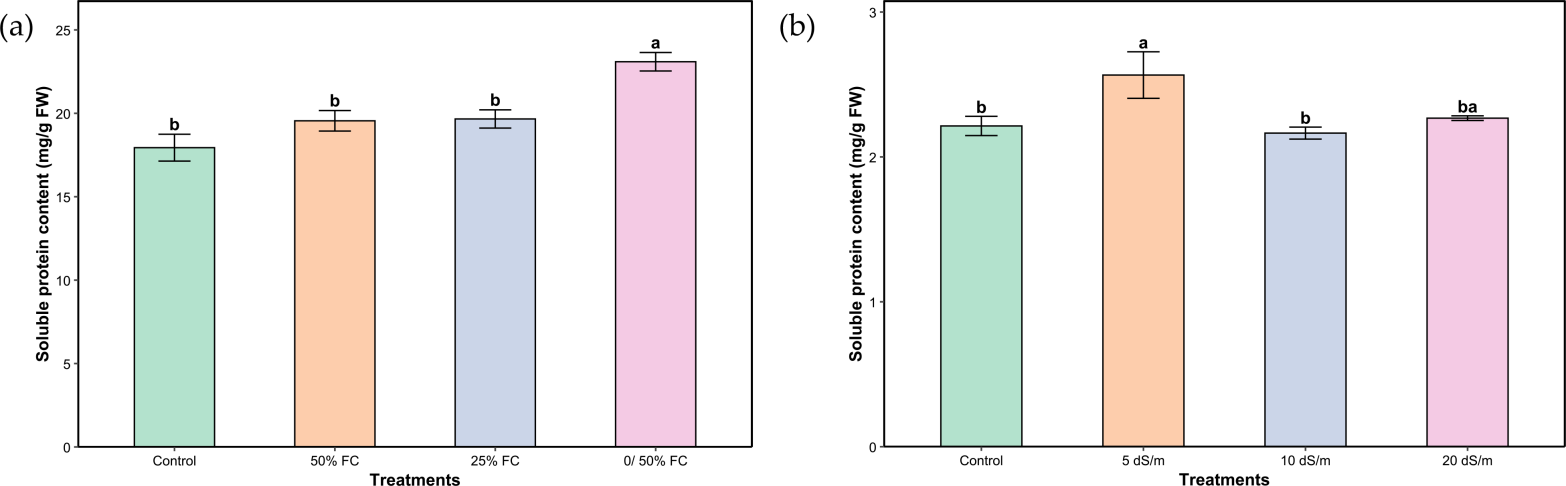
Soluble proteins of aerial parts of *Lavandula coronopifolia* under water **(a)** and salt stress **(b)** treatments. Error bars show the mean ± standard error (n = 9 for water stress and n = 16 for salinity treatments). Significant differences at p < 0.05 level of significance are indicated by different letters above each bar. *0/50% FC: alternating 0% and 50% field capacity weekly. * FW: Fresh weight.

Average soluble protein content in water- and salt-stressed plants increased relative to the control (**Figures 3A, B**). Moderate and severe water stress (50% and 25% FC) did not have a significant impact on soluble protein content. Although, very severe stress (0/50% FC) induced a highly significant increase of 28.71%. Under salt stress, plant response in terms of soluble protein content did not show a statistically significant change across all stress levels. However, moderate stress (5 dS/m) significantly increased the content compared to both the control and severe stress (10 dS/m). The very severe salt stress treatment (20 dS/m) did not show a significant difference from any group, suggesting variable or overlapping effects across treatments.

**Figure 4:**
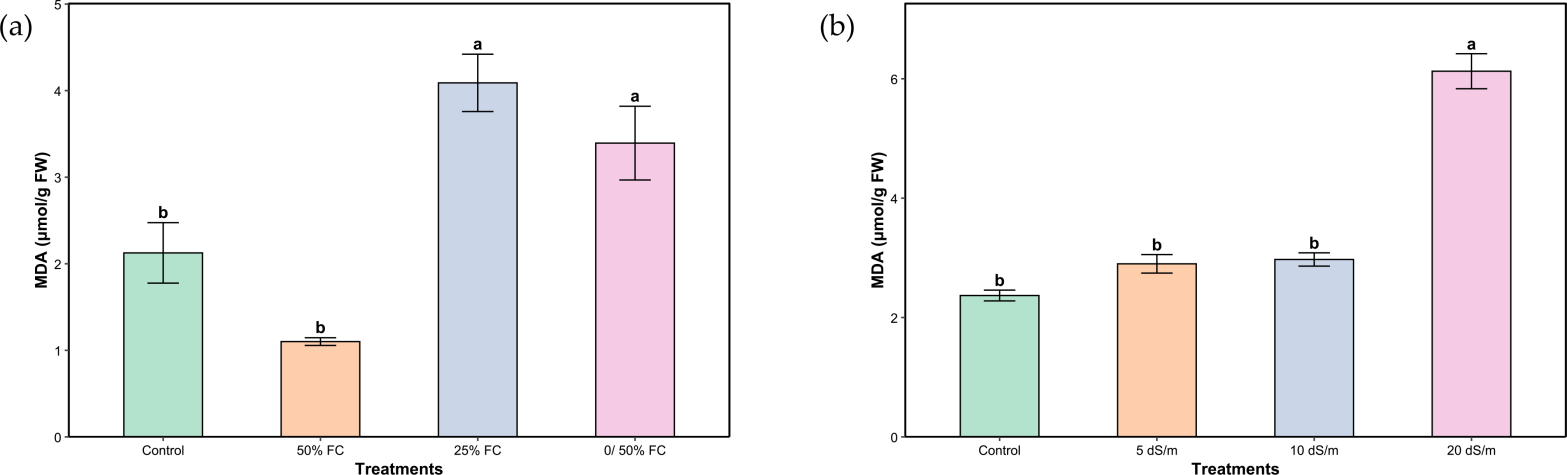
Malondialdehyde of aerial parts of *Lavandula coronopifolia* under water **(a)** and salt stress **(b)** treatments. Error bars show the mean ± standard error (n = 9 for water stress and n = 16 for salinity treatments). Significant differences at p < 0.05 level of significance are indicated by different letters above each bar.*0/50% FC: alternating 0% and 50% field capacity weekly. * FW: Fresh weight.

Under our trial conditions, a significant variation in average MDA was shown under both stresses (**Figures 4A, B**). Although the content was lower in plants irrigated at 50% FC compared to the control, the difference was not statistically significant. However, a progressive increase was observed with increasing stress levels, reaching a 92.92% higher value under severe stress (25% FC). Plants under salinity stress showed a statistically significant response similar to that observed under water stress, with a gradual increase that remained statistically non-significant until the very severe stress level (20 dS/m), where a marked increase of 158.65% was recorded.

### Phytochemical analyses

**Figure 5:**
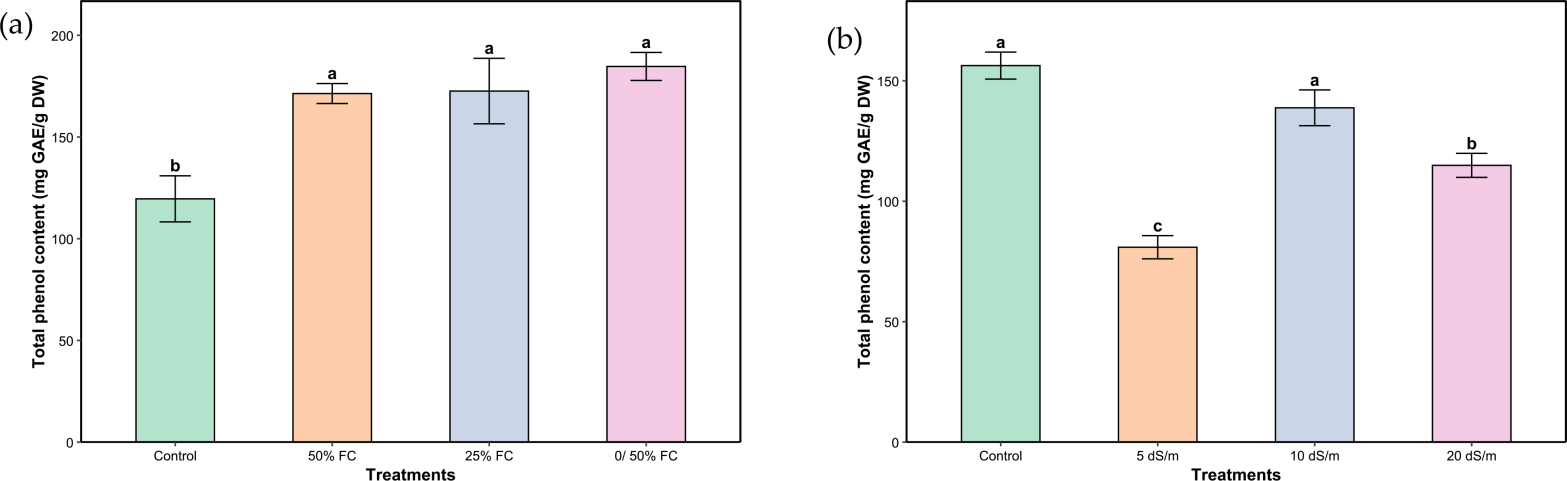
Total phenolic content of aerial parts of *Lavandula coronopifolia* under water **(a)** and salt stress **(b)** treatments. Error bars show the mean ± standard error (n = 9 for water stress and n = 16 for salinity treatments). Significant differences at p < 0.05 level of significance are indicated by different letters above each bar. *0/50% FC: alternating 0% and 50% field capacity weekly. * DW: Dry weight.

The different levels of water and salt stresses had a significant effect on the total phenolic content in the aerial parts of *L. coronopifolia* (**Figures 5A, B**). The highest increase under water stress was observed in plants subjected to very severe stress (0/50% FC), with a 53.66% rise compared to the control. It was followed by severe and moderate stress (25% and 50% FC) with an increase of 43.75% and 42.67%, respectively. In response to salinity, both the control and severe stress (10 dS/m) treatments maintained relatively high concentrations of polyphenols. Moderate and very severe (5 and 20 dS/m) stress treatments led to a significant reduction in phenolic content reaching 48%, 24% and 26.49%, respectively.

**Figure 6:**
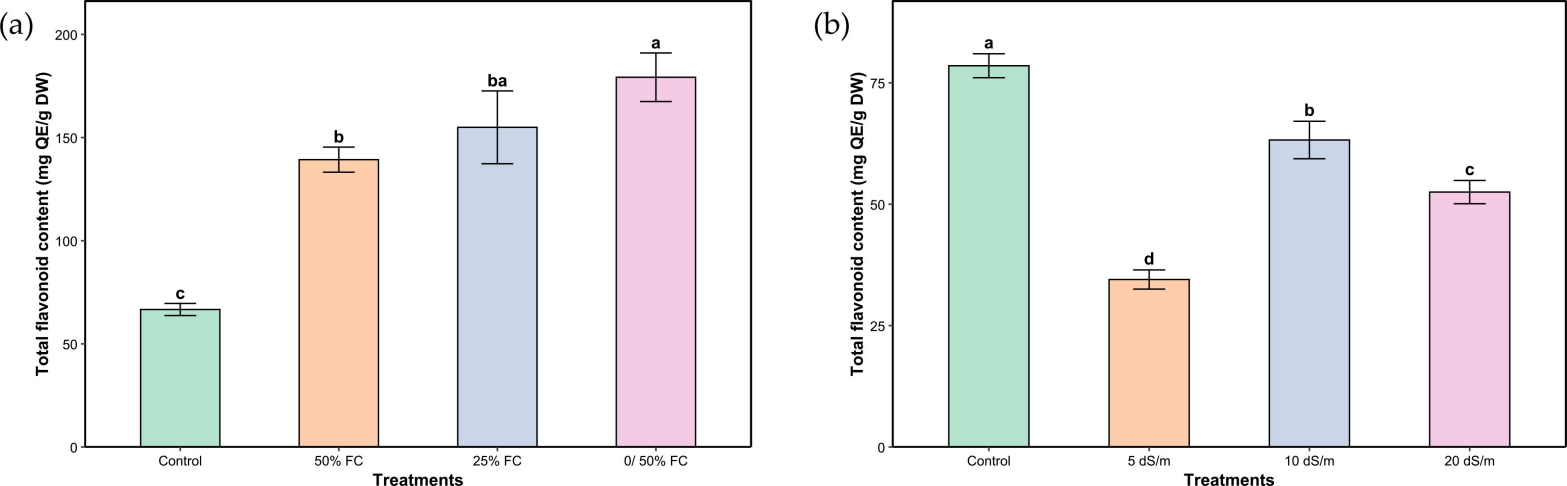
Total flavonoid content of aerial parts of *Lavandula coronopifolia* under water **(a)** and salt stress **(b)** treatments. Error bars show the mean ± standard error (n = 9 for water stress and n = 16 for salinity treatments). Significant differences at p < 0.05 level of significance are indicated by different letters above each bar. *0/50% FC: alternating 0% and 50% field capacity weekly. * DW: Dry weight.

Similarly, total flavonoid content was significantly affected by the applied water and salt stress treatments (**Figures 6A, B**). Total flavonoid content followed the same trends as phenols. Response to water stress exhibited much higher increases, in the order of 107.14%, 132.14% and 167.86%, respectively under moderate, severe and very severe stress. Conversely, under salinity, the highest flavonoid content was observed under control conditions. Exposure to moderate stress led to a marked decrease (56.06%), while severe and very severe stress induced a partial recovery (19.48% and 33.14%, respectively), though still significantly lower compared to the control.

**Figure 7:**
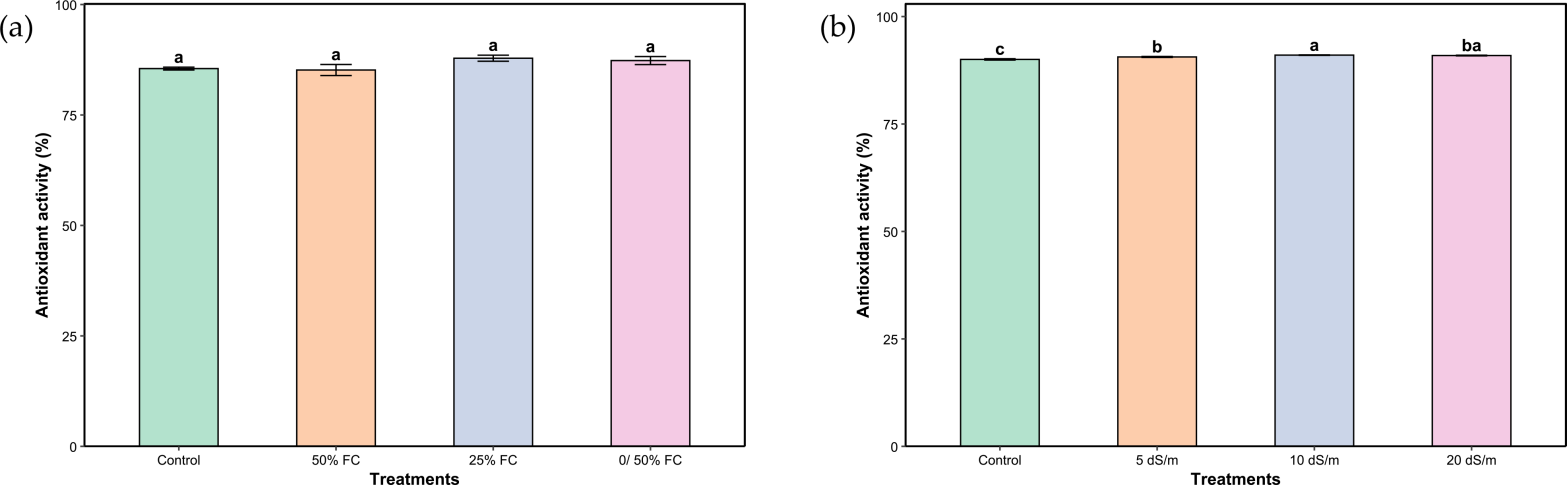
Total antioxidant activity of aerial parts of *Lavandula coronopifolia* under water **(a)** and salt stress **(b)** treatments. Error bars show the mean ± standard error (n = 9 for water stress and n = 16 for salinity treatments). Significant differences at p < 0.05 level of significance are indicated by different letters above each bar. *0/50% FC: alternating 0% and 50% field capacity weekly.

Regarding total antioxidant activity, water stress treatments had no significant effect (**Figure 7A**). In contrast, under salt stress, antioxidant activity displayed modest yet statistically significant differences among treatments (**Figure 7B**). The peak activity was observed at the severe stress level (10 dS/m), marginally exceeding the values under control and moderate stress. Notably, very severe stress did not result in a further enhancement of antioxidant activity.

### Principal component analysis

**Figure 8:**
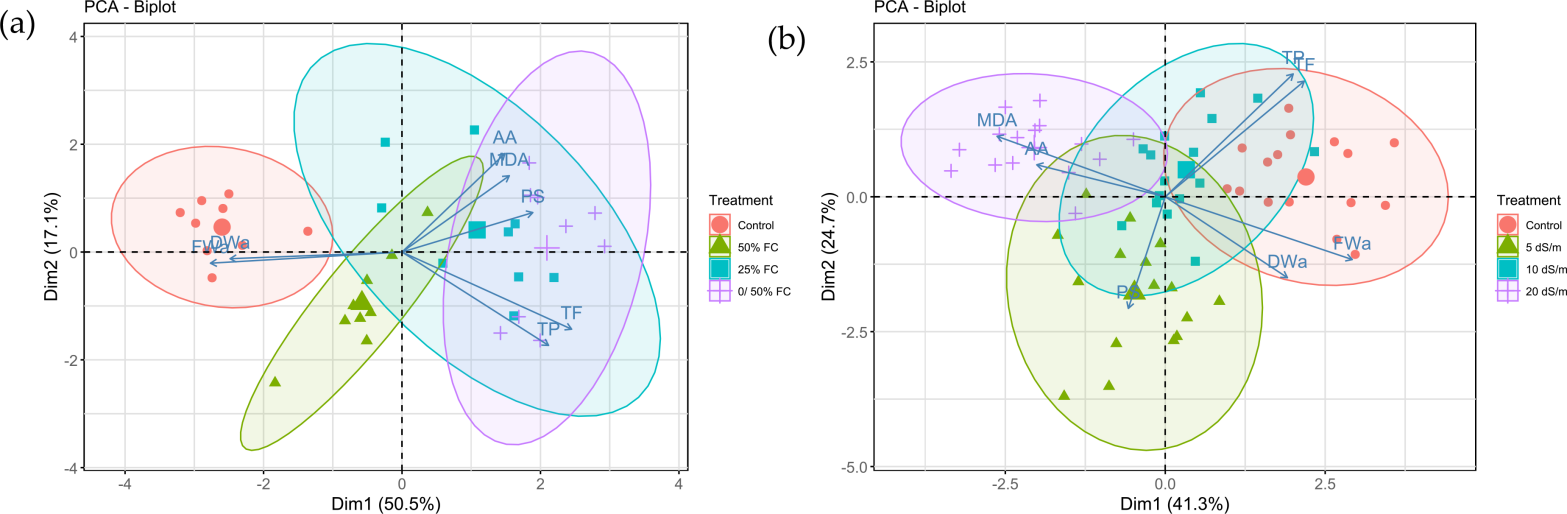
Principal component analysis (PCA) biplot illustrating the variation in morphological, physiological and phytochemical parameters of *Lavandula coronopifolia* under different water **(a)** and salt **(b)** stress treatments.

Principal component analysis (PCA) was performed to explore the multivariate response of the plant to different water stress treatments (**Figure 8A**). The first two principal components (PC1 and PC2) explained a substantial portion of the total variance (67.6%). The PCA biplot revealed a clear separation between the control group and the stressed treatments along the PC1 axis, indicating a distinct morphological profile in non-stressed plants. In contrast, the three water stress treatments (50% FC, 25% FC, and 0/50% FC) clustered together on the opposite side of PC1, suggesting a similar response pattern under reduced water availability.

A second principal component analysis (PCA) was conducted to assess variation among salt treatments (**Figure 8B**). The first two principal components explained a moderate proportion of the total variance (66%). Unlike the PCA for water stress, the treatments showed substantial overlap in the center of the biplot, with no clear separation along either PC1 or PC2. This overlap is consistent with the more variable and less pronounced trends observed in the univariate analyses for several traits under salt stress, indicating that the measured variables did not differentiate the treatments strongly in multivariate space.

The PCA loadings for all variables, showing the contribution of each trait to the principal components, are provided in **Supplementary Table 1** for water stress and **Supplementary Table 2** for salt stress.

### Analysis of individual phenolic and flavonoid compounds

**Table 2** shows how drought and salinity affect the levels of key phenolic and flavonoid compounds in *L. coronopifolia* plants. Under water stress treatments, the concentrations of phenolic and flavonoid compounds in *Lavandula coronopifolia* generally showed an increasing trend, although differences were not statistically significant. In contrast, salt stress had a pronounced inhibitory effect on most compounds. Concentrations of caffeic acid and p-coumaric acid declined significantly at higher salinity levels (10 and 20 dS/m), with near or complete absence of these compounds at 10 dS/m. Similarly, ellagic acid, epicatechin, and naringenin decreased significantly as salinity increased, with their lowest concentrations recorded at 20 dS/m reaching 76.3%, 100% and 81.5% decrease, respectively. These results indicate that salt stress negatively impacts the synthesis or accumulation of key phenolic and flavonoid compounds in *L. coronopifolia*.

**Table 2:** Effects of water stress and salinity on phenolic and flavonoid compound accumulation in aerial parts of *L. coronopifolia*.

Phenolic and flavonoid compound	Water stress				Salt stress			
	Control	50%FC	25% FC	0/50% FC	Control	5 dS/m	10 dS/m	20 dS/m
p-Salicylic acid	27.43 ± 12.94a	19.63 ± 8.22a	20.67 ± 8.42a	25.77 ± 14.07a	1.6 ± 0.25a	0.72 ± 0.73a	0.00 ± 0.00a	0.27 ± 0.28a
Vanillic Acid	0.00 ± 0.00a	0.00 ± 0.00a	0.00 ± 0.00a	0.00 ± 0.00a	0.67 ± 0.06a	0.25 ± 0.15a	0.45 ± 0.17a	0.67 ± 0.07a
Caffeic Acid	289.8 ± 167.04a	458.3 ± 261.82a	517.6 ± 385.29a	369.47 ± 252.69a	3.25 ± 0.28a	1.72 ± 1.00ab	0.00 ± 0.00b	1.05 ± 0.16b
p-Coumaric Acid	73.4 ± 34.64a	158.07 ± 99.27a	120.8 ± 84.62a	58.07 ± 34.09a	2.17 ± 0.49a	1.67 ± 0.87ab	0.00 ± 0.00b	0.00 ± 0.00b
Sinapinic Acid	208.33 ± 208.33a	395.23 ± 274.87a	419.87 ± 419.87a	1298.6 ± 841.67a	45.37 ± 1.22a	56.10 ± 11.87a	41.52 ± 7.53a	27.17 ± 7.90a
Ellagic Acid	33.53 ± 23.53a	60.13 ± 46.78a	63.7 ± 50.84a	57.7 ± 18.22a	5.77 ± 0.28a	2.92 ± 0.60b	1.75 ± 0.18bc	1.37 ± 0.11c
Epicatechin	613.6 ± 349.20a	703.8 ± 427.90a	979.0 ± 506.80a	733.5 ± 462.80a	4.35 ± 0.52a	1.50 ± 0.91b	0.8 ± 0.46b	0.00 ± 0.00b
Naringenin	330.47 ± 190.80a	641.73 ± 641.73a	571.97 ± 425.10a	2441.73 ± 1535.70a	32.60 ± 3.62a	19.77 ± 3.85b	13.42 ± 0.97bc	6.02 ± 1.19c

Values are expressed as means ± standard error (SE) (n = 9 for water stress and n = 16 for salinity treatments). For each parameter and under each type of stress, different letters indicate significant differences at p < 0.05 level of significance.

Overall, drought stress tended to enhance phenolic and flavonoid content, while salt stress reduced their accumulation, highlighting differential metabolic responses of *L. coronopifolia* to these abiotic stresses.

## Discussion

### Effect of water and salt stresses on growth

Water limitation presents a major stress for many plant species, leading to an increase in reactive oxygen species. ROS are naturally produced by plants as byproducts of metabolic processes such as respiration and photosynthesis, and they play key roles in homeostasis and cell signaling (Miller et al., 2008). Their presence in high concentrations triggers the activation of plant defense mechanisms. Plants possess several systems that help reducing the negative impact of external afflictions. These include a range of morphological, physiological, and phytochemical changes. Morphological traits tend to be the most noticeable after stress since plants reallocate their primary metabolism toward defense mechanisms (Munns and Tester, 2008; Caser et al., 2018; Safaei Chaeikar et al., 2020). This growth inhibition is a result of osmotic stress which leads to a series of reactions including reduction of cell turgor, stomata closure, and decrease of cell division (Munns and Tester, 2008). In the present study, the assessment of fresh and dry biomass of *L. coronopifolia* demonstrated that one month of various drought and salt stress regimes application had significant negative effects on plant growth (**Figure 1**; **Figures 2A, B**). The decrease of biomass production was more substantial with the intensity of stress, primarily because of the more pronounced dehydration. Previous studies demonstrated the significant decrease of plant growth under water stress (70% ETo) in other plants from the same genus or family, as it is the case of *Lavandula latifolia* Medik., *Mentha piperita* L. and *Thymus capitatus* Hoffmanns. & Link (García-Caparrós et al., 2019). Similarly, another study revealed that *Origanum majorana* L. shoot fresh weight decreased under water deficit (50% FC) (Farouk and Al-Ghamdi, 2021). Predictably, salinity has a similar effect on plant weight. Various *Lavandula* species exhibited different levels of fresh weight reduction, with *Lavandula multifida* L. presenting the most pronounced decrease reaching 52% at 300 mM NaCl (Plaza et al., 2015). Also, *Salvia hispanica* L. showed a dramatic reduction in fresh weight under severe salt stress conditions (40 and 60 mM NaCl) (Raimondi et al., 2017). These differences in response to both stresses vary depending on the species as well as the ecological conditions.

### Effect of water and salt stresses on physiological parameters

In response to abiotic stresses, plants typically experience an excessive production of reactive oxygen species (ROS), which leads to membrane lipid peroxidation and alters the degree of fatty acid unsaturation (Loreto and Schnitzler, 2010). Malondialdehyde (MDA), a key oxidative stress marker, is accumulated as a by-product of lipid peroxidation and contributes to membrane destabilization and ion leakage (Gapińska et al., 2007). In our study, MDA levels showed distinct patterns under water and salt stress in *L. coronopifolia* (**Figures 4A, B**). Under water stress, the significant increase in MDA concentrations under severe and very severe stress indicates enhanced oxidative damage (Alharbi et al., 2022). This reflects the plants reduced ability to preserve membrane integrity and control ROS accumulation when water availability becomes critically low. The decrease in MDA observed under moderate water stress, however, may indicate an adaptive phase during which antioxidant defenses are activated. Similar drought-induced membrane vulnerability has been reported for *Lavandula angustifolia*, *Thymus vulgaris*, and *T. daenensis* (Emami Bistgani et al., 2017; Mohammadi et al., 2018; Gorgini Shabankareh et al., 2021). For salt stress, MDA increased significantly only under very severe stress, whereas moderate and severe salinity did not differ from the control. This suggests that at low to moderate salinity, *L. coronopifolia* may maintain ionic homeostasis and avoid early oxidative damage. Under very severe salt stress, however, excessive Na^+^ accumulation likely disrupts cellular ion balance, impairs antioxidant systems, and leads to pronounced lipid peroxidation and membrane degradation. A similar rise in MDA under high salinity has been documented in *L. angustifolia* exposed to 100–300 mM NaCl (Szekely-Varga et al., 2020a).

Plants also mitigate stress-induced cellular damage by accumulating osmoprotectants (compatible solutes), including soluble proteins (Blum, 2017). In our study, soluble protein content increased markedly under water stress, especially under very severe conditions (**Figure 3A**). This rise may reflect the synthesis of stress-responsive and protective proteins that stabilize membranes, maintain osmotic balance, and mitigate oxidative damage during dehydration.

In contrast, salt stress induced only moderate changes in soluble protein content (**Figure 3B**). The increase under moderate salinity suggests activation of early osmoregulatory and protective responses. However, the absence of significant changes under severe and very severe salinity may indicate that high ionic toxicity impairs metabolic functions, limiting the plant’s ability to synthesize or accumulate protective proteins.

### Effect of water and salt stresses on phytochemical parameters

Phenols and flavonoids are classes of secondary metabolites with antioxidant activities, playing an essential role in different plant mechanisms including defense system (Talbi et al., 2020), especially in scavenging ROS and reducing oxidative damage (Selmar and Kleinwächter, 2013). Phenolic and flavonoid compounds could be key elements in the enhancement of stress tolerance in plants (Bautista et al., 2016; Sharma et al., 2017; Arora et al., 2018). Increasing contents of both phenols and flavonoids in *L. coronopifolia* were observed under water stress, although the highest mean values above the control plant were reached under very severe stress (**Figure 5A** and **Figure 6A**). The increase of these metabolites indicates an active stimulation of secondary metabolism as part of the plant’s adaptive strategy. Similar results were observed within studies on *Tagetes minuta* L., *Lycium barbarum* L. and *Lavandula stricta* Delile (Polat et al., 2020; Babaei et al., 2021; Gorgini Shabankareh et al., 2021). On the other hand, *Mentha piperita* showed a reduction in both phenols and flavonoids under drought stress (Alhaithloul et al., 2020). Also, a decrease in flavonoids was reported in *Melissa officinalis* L (Radácsi et al., 2016). Under salt stress conditions, total phenolic and flavonoid content showed a differential response to different treatments (**Figure 5B** and **Figure 6B**). Interestingly, under severe stress conditions, total phenol concentrations remained stable and similar to those of the control, suggesting a potential threshold effect beyond which phenolic metabolism is maintained as part of the plant’s defensive strategy. In contrast, both moderate and very severe stress levels led to a reduction in phenol content, possibly reflecting an imbalance between synthesis and degradation processes under suboptimal stress intensities. Flavonoid content, however, showed a consistent decline across all stress treatments relative to the control. This pattern highlights a greater sensitivity of flavonoid biosynthesis to stress intensity, possibly due to its dependence on precursor availability, which may be compromised under prolonged or extreme conditions. Likewise, a study on *Ocimum basilicum* L. reported a decrease in total phenolic and flavonoid contents under up to 100 mM NaCl (Kahveci et al., 2021). On the other hand, *Lavandula angustifolia* plants subjected to salt stress exhibited a slight increase in phenol and flavonoid contents under 100, 200, and 300 mM NaCl treatments (Szekely-Varga et al., 2020a).

Total antioxidant activity of *L. coronopifolia* showed no noticeable variation under water stress, suggesting that the antioxidant defense system remained stable regardless of drought intensity (**Figure 7A**). This stability may reflect an efficient basal antioxidant capacity in wild lavender that is sufficient to cope with water limitation without requiring further upregulation. These findings are aligned with what has been reported by Mokgehle et al (Mokgehle et al., 2017), where *Siphonochilus aethiopicus* (Schweinf.) B.L.Burtt did not display any variation under moderate and severe water stress. In contrast, salt stress induced modest differences among treatments (**Figure 7B**). This indicates that saline conditions, even at moderate levels, can disrupt cellular redox balance to a degree that requires a measurable adjustment in antioxidant defenses.

It is important to note the limitations of this study. The observed changes are correlative, and the underlying causes require further investigation. The experiment was conducted in small pots, and certain measurements, such as substrate pH, conductivity, cation exchange capacity, and Na^+^/K^+^ concentrations, were not performed. These factors should be addressed in future studies to better understand the mechanisms underlying these responses.

## Conclusion

In conclusion, our findings demonstrate that drought and salinity trigger differential metabolic responses in *L. coronopifolia*. While both stresses inhibit growth, they divergently influence secondary metabolism. Drought stress enhanced the accumulation of total phenolics and flavonoids, suggesting an activation of the antioxidant defense system. In contrast, salt stress generally suppressed these compounds, indicating either a disruption of biosynthetic pathways or a shift to alternative tolerance mechanisms. Consequently, controlled drought stress may serve as a viable strategy to enhance the yield of valuable phytochemicals in this species. The potential interactive effects of combined mild stress conditions warrant further investigation to optimize cultivation practices for this resilient, yet vulnerable, medicinal shrub.

## Data Availability

The raw data supporting the conclusions of this article will be made available by the authors, without undue reservation.

## Supplementary material

Supplementary Table 1 Loadings matrix of the PCA performed on plant responses to water stress.

Supplementary Table 2 Loadings matrix of the PCA performed on plant responses to salt stress.
