# Supplementary material for: Water deficit and salinity stresses modulate growth, physiology, and phytochemical composition of Lavandula coronopifolia Poir. grown in pots under controlled conditions
Source: Front Plant Sci. 2026 Jan 7;16:1719460. doi: 10.3389/fpls.2025.1719460 (PMC12819654; doi:10.3389/fpls.2025.1719460)
Supplement: Supplementary Table 1 — Loadings matrix of the PCA performed on plant responses to water stress. [file Table1.docx]

## Supplementary data

Table 1: Loadings matrix of the PCA performed on plant responses to water stress

|  | **Dim.1** | **Dim.2** | **Dim.3** | **Dim.4** | **Dim.5** |
| --- | --- | --- | --- | --- | --- |
| Fresh weight of aerial parts | -0.91241 | -0.06828 | 0.171545 | -0.01781 | 0.246771 |
| Dry weight of aerial parts | -0.81907 | -0.04087 | 0.254737 | 0.08378 | 0.45511 |
| Soluble protein content | 0.623108 | 0.24155 | -0.49687 | 0.298953 | 0.463533 |
| MDA | 0.510929 | 0.466749 | 0.562513 | 0.446083 | -0.06088 |
| Total phenol content | 0.697968 | -0.57127 | 0.243019 | -0.10349 | 0.227462 |
| Total flavonoid content | 0.807977 | -0.47206 | 0.170165 | -0.01295 | 0.096438 |
| Antioxidant activity | 0.489967 | 0.602856 | 0.163804 | -0.5697 | 0.211273 |

Table 2: Loadings matrix of the PCA performed on plant responses to salt stress

|  | **Dim.1** | **Dim.2** | **Dim.3** | **Dim.4** | **Dim.5** |
| --- | --- | --- | --- | --- | --- |
| Fresh weight of aerial parts | 0.87611 | -0.34871 | -0.11439 | -0.0313 | -0.14738 |
| Dry weight of aerial parts | 0.572421 | -0.44959 | -0.34964 | 0.38245 | 0.4404 |
| Soluble protein content | 0.599327 | 0.68239 | 0.186539 | 0.224159 | -0.04987 |
| MDA | 0.651354 | 0.64245 | 0.134685 | 0.238367 | -0.006 |
| Total phenol content | -0.60203 | 0.177605 | -0.53667 | 0.444245 | -0.34582 |
| Total flavonoid content | -0.17297 | -0.62013 | 0.5914 | 0.454076 | -0.17165 |
| Antioxidant activity | -0.78819 | 0.336094 | 0.152205 | 0.171431 | 0.41495 |
